# High-mobility group box 1 protein is implicated in advanced glycation end products–induced vascular endothelial growth factor A production in the rat retinal ganglion cell line RGC-5

**Published:** 2012-04-05

**Authors:** Jong-Jer Lee, Chang-Chun Hsiao, I-Hui Yang, Ming-Huei Chou, Chia-Lin Wu, Yin-Chu Wei, Chih-Hsin Chen, Jiin-Haur Chuang

**Affiliations:** 1 Department of Ophthalmology, Kaohsiung Chang Gung Memorial Hospital and Chang Gung University College of Medicine, Kaohsiung, Taiwan; 2 Department of Surgery, Division of Pediatric Surgery, Kaohsiung Chang Gung Memorial Hospital and Chang Gung University College of Medicine, Kaohsiung, Taiwan; 3 The Graduate Institute of Clinical Medical Sciences, Chang Gung University College of Medicine, Kaohsiung, Taiwan

## Abstract

**Purpose:**

High-mobility group box 1 protein (HMGB1) has been reported to be a potent proangiogenic factor induced by inflammatory stress. In this study, we explore the role of HMGB1 in advanced glycation end products (AGEs)–induced vascular endothelial growth factor A (VEGF-A) production in rat retinal ganglion cell line 5 (RGC-5) cells.

**Methods:**

The VEGF-A protein and mRNA levels in conditioned medium of RGC-5 cells incubated with AGE-modified BSA (AGE-BSA) were examined with real-time PCR and enzyme-linked immunosorbent assay (ELISA), and BSA-treated cells were used as controls. The expression of HMGB1, c-Jun N-terminal kinase (JNK), extracellular-signal-regulated kinase (ERK), and p38 mitogen-activated protein kinase (p38 MAPK) was assessed with immunofluorescence and western blot analysis. Reactive oxidative species (ROS) were detected with flow cytometry measurements of peroxide-dependent oxidation of 2′-7′-dichlorofluorescein-diacetate (DCFH-DA). N-Acetyl-L-cysteine (NAC), glycyrrhizin (GZ), and SP600125 were used to block ROS, HMGB1, and JNK, respectively.

**Results:**

Compared with the BSA controls, the RGC-5 cells incubated with AGE-BSA showed a dose- and time-dependent increase in *VEGF-A* mRNA and VEGF-A protein secretion in the supernatant, with the highest levels achieved at 24 h. AGE-BSA stimulated a significant release of HMGB1 in the supernatant and a significant increase of intracellular ROS production at 3 h. NAC blocked HMGB1 production in a dose-dependent manner. Blocking with GZ, NAC, and JNK significantly suppressed AGE-induced VEGF-A production.

**Conclusions:**

HMGB1 is implicated in the production of VEGF-A in retinal ganglion cell line-5 (RGC-5). Blocking HMGB1, ROS, or the JNK pathway may attenuate VEGF-A production, suggesting HMGB1 and related signaling molecules play a role in diabetic retinopathy.

## Introduction

Diabetic retinopathy is one of the leading causes of vision loss in patients under 65 years of age [1]. Diabetes causes retinal microvasculopathy associated with pericyte cell death, microaneurysms, abnormal vascular permeability, and macular edema. Long-term microvasculopathy results in retinal hypoxia and subsequent neovascularization with abnormal blood vessels proliferating into the vitreal cavity [2,3].

Glycation, the result of a protein or lipid molecule bonding with sugar molecules, is a consequence of the aging process. The results of a chain of chemical reactions after the initiation of glycation are now referred to as advanced glycation end products (AGEs), which can contribute to the accelerated micro- and macrovasculopathy observed in diabetes [4]. AGEs induce vascular endothelial growth factor A (VEGF-A) production via the receptor for advanced glycation end products (RAGE) and activation of nicotinamide adenine dinucleotide phosphate (NADPH) oxidase or protein kinase C (PKC)-alpha in mesangial cells [5]. In addition to AGEs, high-mobility group box protein 1 (HMGB1) is another ligand of RAGE [6]. HMGB1 is present in the nucleus of all mammalian cells, where this protein induces structural and transcriptional activities under physiologic conditions [7-9]. However, HMGB1 is also implicated as an important endogenous danger signaling molecule and amplifies the activities of immunostimulatory molecules in a synergistic manner [10,11].

Presently, the clinical treatment for diabetic retinopathy is limited to pan-retinal photocoagulation and vitrectomy for late proliferative disease and anti-VEGF therapy for controlling macular edema that impairs vision. Identifying new strategies for treatment before the late stage of retinopathy is desirable. HMGB1 has been identified as a potent proangiogenic stimulus in experimental studies [12,13], and its roles in various retinal diseases are being elucidated [14-18]. In this study, we investigate the role of HMGB1 in retinal ganglion cell line 5 (RGC-5) cells, a source of retinal VEGF-A [19,20]. We expect our findings will provide clues for future management of diabetic retinopathy.

## Methods

### Chemical and instrument suppliers

Dulbecco’s phosphate buffered saline (DPBS) was purchased from Hyclone (Logan, IL). Dulbecco’s modified Eagle’s medium (DMEM) and fetal bovine serum (FBS) were from Invitrogen-GIBCO (Carlsbad, CA). Kits for RNA extraction were from Qiagen (Venlo, the Netherlands). Primers for quantitative real-time PCR were from Genomics (Taipei, Taiwan). Protein extraction buffer was from GE Healthcare (Little Chalfont, UK). The Subcellular fraction extraction kit S-PEK was from Merck4Bioscences (Darmstadt, Germany). The BCA Protein Assay Kit was from Thermo Scientific (Waltham, MA). ECL western blotting detection reagents and polyvinylidene fluoride membrane were from Millipore (Billerica, MA). The Rat VEGF-A enzyme-linked immunosorbent assay (ELISA) assay kit was from Peprotech (Rocky Hill, NJ). The Cell Proliferation Kit II (XTT) and the Cytotoxicity Detection Kit (LDH) were from Roche (Penzburg, Germany). Anti-p38 (rabbit polyclonal) and anti-glyceraldehyde 3-phosphate dehydrogenase (GAPDH; mouse monoclonal) were from Abcam (Cambridge, UK). Anti-HMGB1 (rabbit monoclonal), antibeta tubulin (rabbit monoclonal), anti-phospho-p38 (rabbit monoclonal), and anti-JNK2 (rabbit monoclonal) were from Epitomics (Burlingame, CA). Anti-phospho-ERK (rabbit monoclonal), anti-ERK, and anti-phospho-JNK (mouse monoclonal) were from Cell Signaling (Beverly, MA). Anti-RAGE (rabbit polyclonal) and anti-Toll-like receptor 4 (anti-TLR4; mouse monoclonal) were from Santa Cruz Biotechnology (Santa Cruz, CA). Alexa Fluor 488 and 594 conjugated secondary antibodies and 4',6-diamidino-2-phenylindole (DAPI) were from Invitrogen (Carlsbad, CA). Mitogen-activated protein kinase (MAPK) inhibitors including SB230580, SP600125, PD98059, and U0126 were from Cayman (Ann Arbor, MI). N-Acetyl-L-cysteine and glycyrrhizin (GZ) were from Sigma-Aldrich (St. Louis, MO). AGE-BSA and BSA for controlled experiments were from Biovision (Mountain View, CA). The Sunrise microplate reader for ELISA was from TECAN (Männedorf, Switzerland). Flow cytometry was performed with FACSCalibur (BD Biosciences, Franklin Lakes, NJ). ECL western blotting analysis was performed with the Molecular Imager Versadoc MP4000 from Bio-Rad (Hercules, CA). Real-time PCR analysis was performed with the LightCycler® 480 Real-Time PCR System from Roche (Penzburg, Germany). The fluorescence microscope Eclipse 800 was from Nikon (Tokyo, Japan).

### Cell culture and treatment with advanced glycation end products or inhibitors of high-mobility box 1 protein and mitogen-activated protein kinase signaling pathways

RGC-5 (courtesy of Dr. Chi-Chun Lai) was obtained from Dr. Neeraj Agarwal, North Texas Health Science Center, Fort Worth, TX. The cell stained positively with anti-neurofilament-M (NF-M) antibody (Cell signaling, Beverly, MA). RGC-5 cells were cultured in DMEM with low glucose and 10% FBS and 1% penicillin/streptomycin mixture at 37 °C in a humidified atmosphere of 5% of CO_2_. For most experiments, 2.5×10^6^ cells were plated in 60-mm dishes (430166; Corning, Lowell, MA). For ELISA assays, 2×10^4^ cells were plated on a 96-well plate (167008; Nunc, Roskilde, Denmark). For immunofluorescence studies, 2.5×10^5^ cells were seeded on a coverslip and mounted on a 24-well plate (142475; Nunc). After sub-confluent cell growth, the medium was replaced by DMEM with 0.5% FBS for 4 h before treatment with AGE-BSA (Biovision). AGE-BSA (200 μg/ml) was added to the culture medium for specific time intervals for individual experiments, and cells treated with BSA (200 μg/ml; Biovision) were used as controls. To study the effects of blocking HMGB1 with glycyrrhizin (GZ), or blocking the MAPK pathway with specific MAPK inhibitors (Cayman), including SB230580, SP600125, PD98059, or U0126, the individual inhibitor was premixed in the medium for 1 h before AGE-BSA was added.

### Protein collection

After treatment, the protein in the supernatant was collected using previously described protocols [21]. Briefly, cell-conditioned medium was centrifuged at 250× g for 10 min at 4 °C to separate detached cells and then harvested carefully and filtered through Millex-GP (Millipore) to remove cell debris and macromolecular complexes. Samples were then concentrated with Amicon Ultra–10,000 NMWL (Millipore) according to the manufacturer’s instructions. After the protein was quantified with the BCA kit, an equal amount of protein derived from the supernatant was loaded for western blot analysis.

### Cell viability assays

The effect of AGE-BSA or BSA on RGC-5 cell viability was measured with a cell proliferation kit (XTT), and cytotoxicity was assessed using a lactate dehydrogenase (LDH) activity assay kit. Cells (2×10^4^) were seeded on a 96-well flat bottom plate (167008; Nunc). After 24-h incubation with either AGE-BSA or BSA, cells were treated with reagents for the XTT assay according to the manufacturer’s instructions (Roche). The percentage of viable cells was compared to that of sterile DPBS-treated cells. To assess the cytotoxic effect of AGE-BSA on RGC-5 cells, we used an LDH assay kit and followed the protocol provided by the manufacturer (Roche). Cells treated with the lysis solution were considered 100% damaged and used as a positive control. The results of both assays were determined based on optical density detected with an ELISA reader (TECAN). The statistical analysis was based on the results of six independent experiments.

### Western blotting for whole cell and fractionated subcellular proteins

After treatment, whole-cell lysates of RGC-5 cells were obtained by washing with cold DPBS and lysed using protein extraction buffer (28–9412–79; GE Healthcare) with a protease inhibitor (Sigma-Aldrich). When subcellular fractionation of protein was required, proteins from the cytosol and nucleus were prepared using S-PEK (Merck Biosciences) according to the manufacturer’s instruction [22]. Proteins derived from subcellular fractions, total cell lysates, or concentrated cell culture supernatant were resolved with sodium dodecyl sulfate–PAGE (4% stacking and 10% resolving gel) and transferred onto a polyvinylidene fluoride membrane (Millipore). Membranes were blocked with SuperBlock blocking buffer (Thermo Scientific). Blots were probed overnight at 4 °C with 1:200 to 1:2,000 of diluted primary antibodies specific for the individual target protein, and then incubated with 1:5,000 diluted horseradish peroxidase–linked secondary antibodies (Jackson ImmunoResearch, West Grove, PA). Images were captured with the ECL imaging system (Bio-Rad). Statistical analysis was based on the results of four independent experiments.

### RNA expression analysis with real-time polymerase chain reaction

Total RNA was extracted from cells using the RNeasy Mini Kit (Qiagen). After quantification, total RNA (1 μg) was used to synthesize cDNA by reverse transcription with oligo(dT)15 primer, Moloney Murine Leukemia Virus reverse transcriptase, and deoxynucleoside triphosphate mixture (Promega, Madison, WI) according to the manufacturer’s recommendations. Quantitative real-time PCR (qPCR) was performed with the Light-Cycler Real-Time PCR System (Roche) to study the expression of *VEGF-A* using SYBR green assays. The sequences of the forward and reverse primers of *VEGF-A* were 5′-CAG CTA TTG CCG TCC AAT TGA-3′ and 5′-CCA GGG CTT CAT CAT TGC A-3′, respectively [23]. The expression levels of β-actin (*Actb*) were used as controls (forward primer, 5′-AGG CCC CTC TGA AAC CTA AG-3′, and reverse primer, 5′-CAA CAC AGC CTG GAT GGC TAC-3′). Analysis was based on the results of three independent experiments.

### Enzyme-linked immunosorbent assay for vascular endothelial growth factor A

After subconfluent growth, RGC-5 cells were starved from serum for 4 h and stimulated by 24-h incubation with fresh media containing different concentrations of AGE-BSA (0, 100, 200, 500, and 1,000 μg/ml). The VEGF-A released into the culture medium was collected and quantified after treatment with AGE-BSA with or without specific inhibitors. The results were compared with those of the control BSA at equal concentrations according to the instruction provided by the manufacturer (Peprotech). Plates were read at 450 nm using a microplate spectrophotometer (TECAN). Analysis was based on the results of eight independent experiments.

### Reactive oxidative species measurement

Intracellular ROS formation after treatment of the RGC-5 cells with AGEs was evaluated using peroxide-dependent oxidation of 2′-7′-dichlorofluorescein-diacetate (DCFH-DA). DCFH-DA is cleaved intracellularly by nonspecific esterases to form DCFH, which is further oxidized by ROS to form the fluorescent compound DCF. DCFH-DA (Sigma-Aldrich) working solution was added directly to the medium to attain a concentration of 2 μM; and then incubated for 30 min at 37 °C. Cells were harvested and then washed once, resuspended in DPBS, and kept on ice for immediate detection with flow cytometry (FACSCalibur; BD Biosciences). Analysis was based on the results of three independent experiments.

### Immunofluorescence analysis of RGC-5 cells after treatment with advanced glycation end products–BSA

Cells were seeded and cultured on glass coverslips precoated with 0.01% poly-L-lysine (Sigma-Aldrich) for 1 h. After the cells were treated with AGE-BSA for 24 h, they were fixed with 4% paraformaldehyde for 30 min and then treated with 0.1% Triton X-100 for 10 min at room temperature. Blocking was achieved with PBS containing 5% normal goat serum for 1 h at room temperature. Cells were then incubated with an anti-HMGB1 antibody overnight at 4 °C, followed by incubation with Alexa Fluor 594 conjugated secondary antibody and DAPI for nuclear staining. Coverslips were mounted on slides using fluorescence mounting medium (Dako, Glostrup, Denmark). Fluorescence image capture and analysis were performed with a fluorescence microscope (Nikon).

### Statistical analysis

Data are shown as means±SD or as a percentage. The Statistical Package for Social Science program (SPSS for Windows, version 13.0; SPSS, Chicago, IL) was used for statistical analysis. A one-way ANOVA was used for comparing the differences between groups. The Mann–Whitney U test was used when normal distribution of results was not observed, and p values less than 0.05 were considered statistically significant.

## Results

### Advanced glycation end products induce vascular endothelial growth factor-A expression and release in culture media in RGC-5 cells

**Figure 1:**
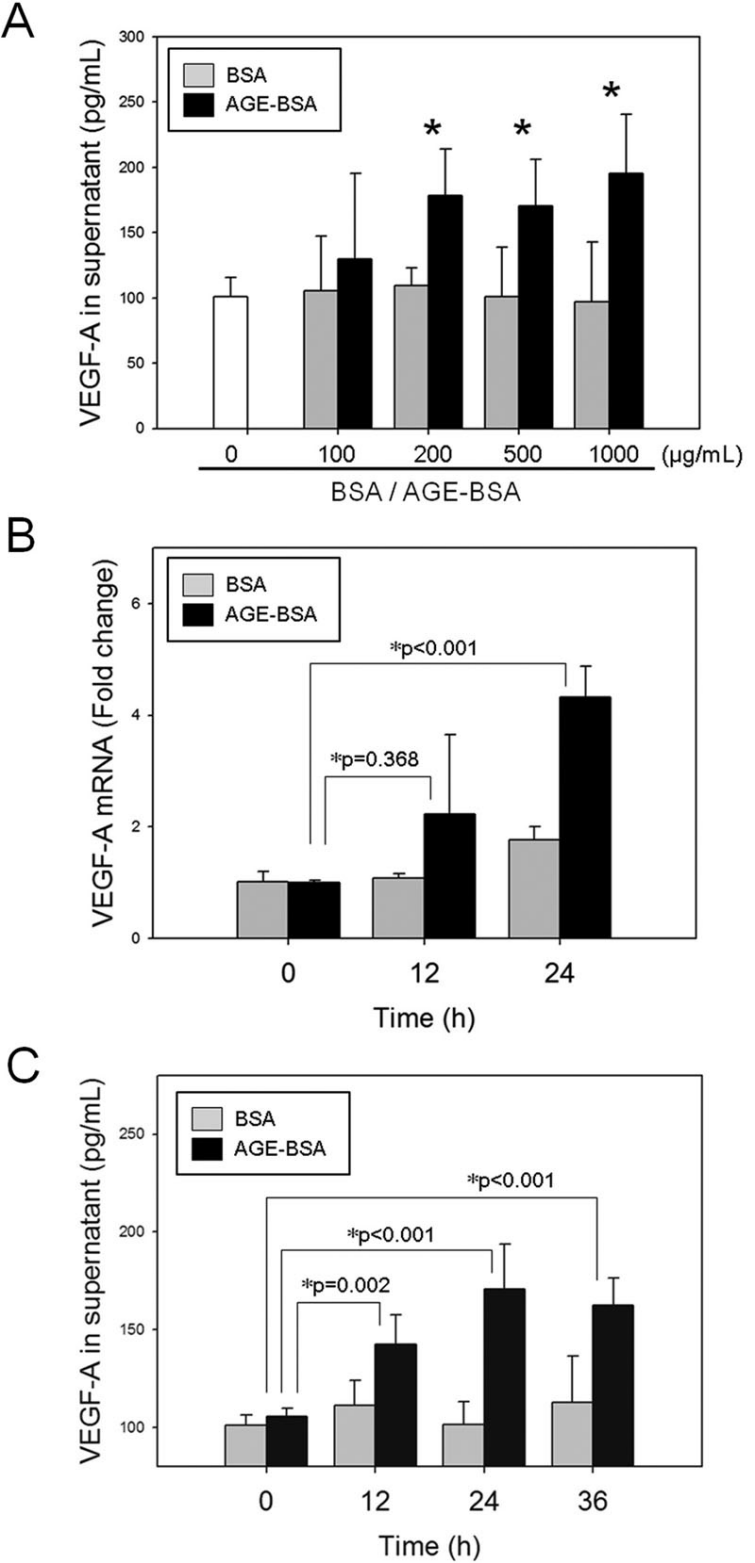
Advanced glycation end products–BSA (AGE-BSA) treatment results in vascular endothelial growth factor (VEGF)-A upregulation in retinal ganglion cell (RGC)-5 cells. **A**: The VEGF-A concentration in the media of RGC-5 cells treated with 100 to 1,000 μg/ml of AGE-BSA for 24 h was detected with enzyme-linked immunosorbent assay (ELISA). (n=8, * p<0.05, between AGE-BSA and BSA control). **B**: The change in the VEGF-A mRNA levels of RGC-5 cell was detected with real-time qPCR after incubation with AGE-BSA (200 μg/ml) for 12 to 24 h (n=3). **C**: VEGF-A concentration in conditioned media increased over time after incubation of RGC-5 cells with 200 μg/ml of AGE-BSA (n=8).

The VEGF-A protein secreted into the culture media was quantified using a VEGF-A ELISA kit (Peprotech). A dose-dependent increase in VEGF-A in the media was found in RGC-5 cells treated for 24 h with increasing concentration of AGE-BSA (from 100 to 1,000 μg/ml), when compared with the VEGF-A level in the BSA control (130.0±65.3 versus 105.2±42.0 pg/ml at 100 μg/ml, 178.4±35.4 versus 105.0±14.4 pg/ml at 200 μg/ml, 171.7±37.9 versus 101.0±32.4 pg/ml at 500 μg/ml, and 195.4±44.9 versus 97.1±45.5 pg/ml at 1,000 μg/ml, respectively; one-way ANOVA with post hoc Bonferroni test, p=1.000, p=0.014, p=0.035, and p=0.025, respectively, Figure 1A). Elevation of *VEGF-A* mRNA levels in RGC-5 cells started 12 h after incubation with AGE-BSA (200 μg/ml) and reached 4.33±0.54 fold expression over baseline values at 24 h (p<0.001, Figure 1B). A time-dependent increase in VEGF-A concentration was detected in the medium after treatment of RGC-5 cells with AGE-BSA for 12, 24, and 36 h (p=0.002, p<0.001, and p<0.001, respectively, Figure 1C).

### Advanced glycation end products induce translocation and release of high-mobility box 1 protein from RGC-5 cells

**Figure 2:**
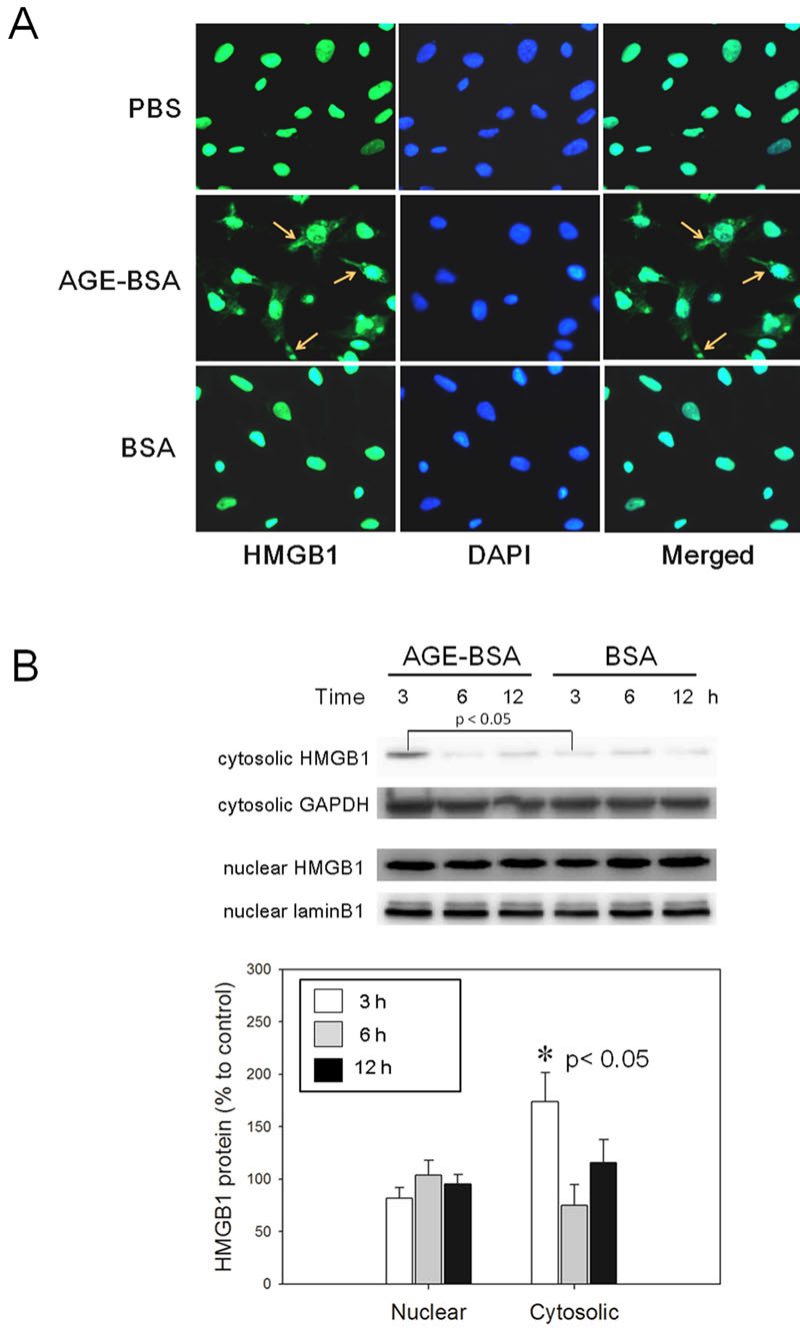
The distribution of high-mobility group box protein 1 (HMGB1) in retinal ganglion cell (RGC)-5 cells changed following treatment with advanced glycation end products–BSA (AGE-BSA). **A**: Immunofluorescence photographs (400× magnification) show that HMGB1 appears in the cytoplasm (arrow) of RGC-5 cells at 3 h after treatment with 200 μg/ml of AGE-BSA, while HMGB1 remains in the nucleus of RGC-5 cells treated with either BSA (200 μg/ml) or PBS. The left column shows the distribution of HMGB1 protein with green fluorescence. The central column shows the nucleus stained with 4',6-diamidino-2-phenylindole (DAPI; blue), and the right column shows merged pictures. **B**: Subcellular fractionation of proteins shows a significant increase of HMGB1 levels in the cytosol of RGC-5 cells after incubation with AGE-BSA for 3 h (n=4, p<0.05, BSA-treated cells were used as control). The level of HMGB1 in the nucleus was not different between groups over time.

**Figure 3:**
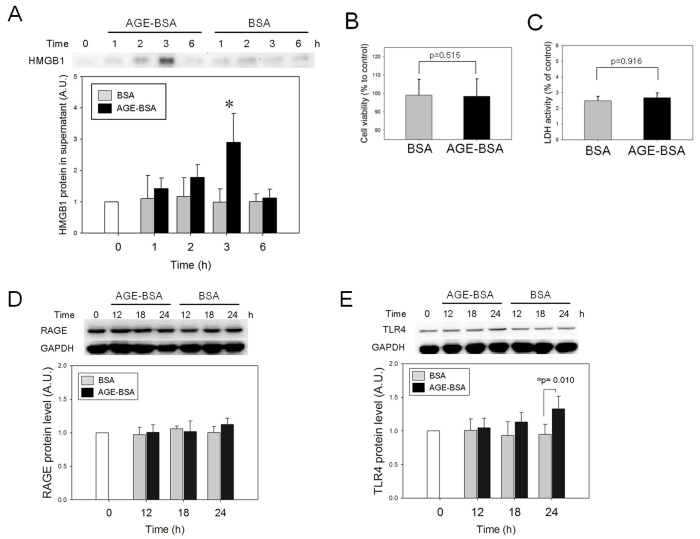
Advanced glycation end products–BSA (AGE-BSA) treatment results in the release of high-mobility group box protein 1 (HMGB1) from retinal ganglion cell (RGC)-5 cells. **A**: HMGB1 in the culture media after treatment of RGC-5 cells with 200 μg/ml of AGE-BSA or BSA alone was detected by western blot analysis. The release of HMGB1 into the media reached significance at 3 h after treatment with AGE-BSA, compared to the BSA control (n=4, * p<0.05; A.U.represents arbitrary unit). **B**: XTT test and (**C**) lactate dehydrogenase (LDH) assay conducted after incubation of RGC-5 cells with 200 μg/ml of AGE-BSA or BSA alone for 24 h. No significant difference was found between groups (n=6, p=0.515 for XTT and p=0.916 for the LDH test). **D**: RAGE and (**E**) TLR4 protein levels were assayed with western blot analysis. The levels of RAGE in the RGC-5 were not different between groups over time. However, a mild but significant increase in the TLR4 protein was detected after AGE-BSA treatment at 24 h (n=5, p=0.010).

Immunofluorescence studies demonstrated that HMGB1 appeared in the cytoplasm after 3 h of incubation with AGE-BSA. Meanwhile, HMGB1 remained localized in the nucleus in control RGC-5 cells treated with BSA or DPBS only (Figure 2A). Significant increases in cytosolic HMGB1 in RGC-5 cells were detected with 3 h of AGE-BSA (200 μg/ml) when compared with the BSA treatment. Nuclear HMGB1 levels remained relatively the same during 3 to 12 h of AGE-BSA treatment. Western blot analysis of intracellular protein of RGC-5 showed that AGE-BSA induced a significant change in the cytosolic HMGB1 by 173.7%±27.6% at 3 h (p=0.029), which then decreased to 81.0%±14.6% at 6 h (p=0.686) and 105.3%±20.7% at 12 h (p=0.343) when compared with the BSA-treated cells. AGE-BSA caused insignificant changes in nuclear HMGB1 by 83.3%±15.1%, 97.1±24.0%, and 96.8%±14.1% (p=0.275, p=0.827, and p=0.827, respectively) at 3, 6, and 12 h, comparing to BSA-treated cells (Figure 2B). Western blot analysis showed a significant increase in HMGB1 in the culture medium of RGC-5 cells incubated with AGE-BSA for 3 h (p<0.005) but not at any other time points (Figure 3A). The viability of the RGC-5 cells assayed with the XTT test and the cytotoxicity assessed with LDH showed no significant difference between the AGE-BSA treated and control groups (Figure 3B,C).

The expressions of RAGE and TLR4 were also assessed with western blot analysis. The RAGE protein levels at 12, 18, and 24 h compare to the baseline in AGE-BSA (200 μg/ml) treated RGC-5 cells were 98.6%±12.5%, 107.4%±2.7%, and 112.1%±9.4%, respectively, and 96.8%±12.6%, 101.9%±15.6%, and 101.3%±6.3%, respectively, in the BSA-treated cells (Figure 3D). The difference between treatments was not significant (p=0.699, p=0.413, and p=0.114 for 12, 18, and 24 h, respectively). The TLR4 protein levels at 12, 18, and 24 h compared to the baseline in the RGC-5 cells treated with AGE-BSA (200 μg/ml) were 104.5%±14.4%, 113.6%±14.1%, and 132.6%±19.0%, respectively, and 101.1%±17.0%, 93.4%±20.4%, and 94.6%±15.4%, respectively, in the BSA-treated cells (Figure 3E). A significant increase in the TLR4 protein was detected at 24 h after the AGE-BSA treatment (p=0.931, p=0.111, and p=0.010 for 12, 18, and 24 h, respectively).

### Reactive oxidative species generation in RGC-5 cells treated with advanced glycation end products and the reverse of high-mobility box 1 protein secretion with antioxidant N-acetyl-L-cysteine

**Figure 4:**
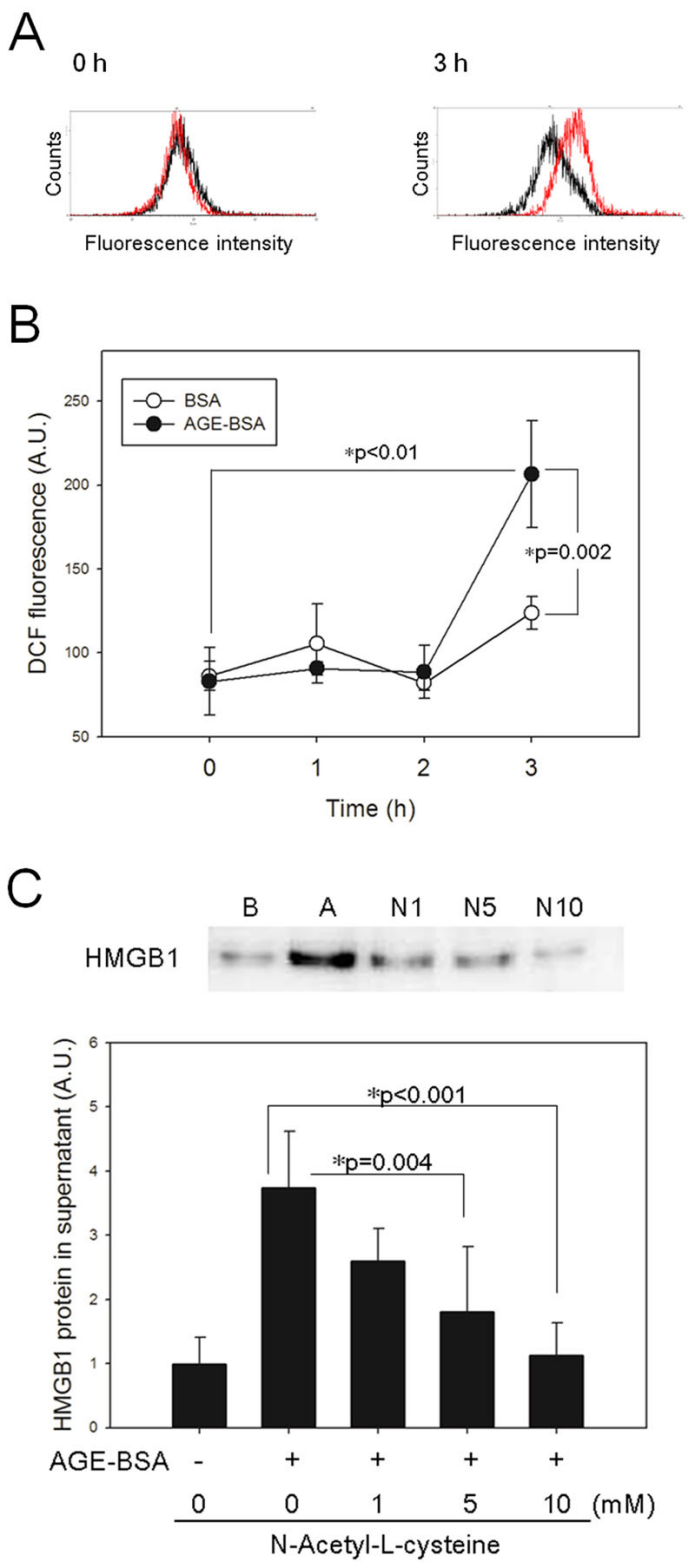
Reactive oxidative species (ROS) could be the trigger for release of high-mobility group box protein 1 (HMGB1) in retinal ganglion cell (RGC)-5 cells. **A**: Flow cytometric histogram shows an increase in ROS at 3 h in RGC-5 cells treated with advanced glycation end products–BSA (AGE-BSA; red) or BSA alone (black). **B**: Histogram shows that DCF fluorescence changes with time (n=3). **C**: Pretreatment of RGC-5 cells with the antioxidant N-acetyl-L-cysteine (NAC) reduces the AGE-BSA-induced HMGB1 protein levels in the supernatant at 3 h in a dose-dependent manner. Abbreviations: **A**, AGEs; **B**, BSA; N1, N5, and N10: RGC-5 cells pretreated with NAC at concentrations of 1, 5, and 10 mM respectively (n=4).

ROS production in the RGC-5 cells treated with AGE-BSA was evaluated using a peroxide-dependent oxidation of DCFH-DA. A significant increase in ROS from baseline was observed in the RGC-5 cells at 3 h after incubation with AGE-BSA, compared with the BSA control (249.1%±38.4% and 153.9%±7.0%, respectively, p=0.002, Figure 4 A,B). Pretreatment of RGC-5 cells with 1, 5, and 10 mM NAC, a known antioxidant [24,25], significantly suppressed the elevation of HMGB1 in a dose-dependent manner (p=0.215, p=0.004, and p<0.001, respectively, Figure 4C).

### c-Jun N-terminal kinase phosphorylation is induced in RGC-5 cells treated with advanced glycation end products and is blocked by a high-mobility box 1 protein inhibitor

**Figure 5:**
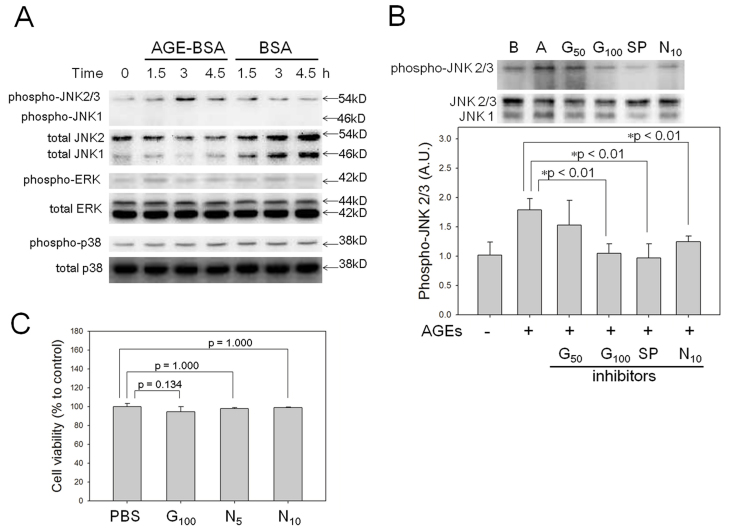
Advanced glycation end products–BSA (AGE-BSA) treatment induces c-Jun N-terminal kinase (JNK)2/3, and JNK2/3 induction is reversed by a high-mobility group box protein 1 (HMGB1) blocker. **A**: The phosphorylations of JNK, extracellular-signal-regulated kinase (ERK), and p38 in mitogen-activated protein kinase (MAPK) signaling in retinal ganglion cell (RGC)-5 cells treated with 200 μg/ml of AGE-BSA for more than 3 h were detected by western blot analysis. Only phospho-JNK2/3, but not phospho-ERK or phospho-p38, significantly increased at 3 h after treatment with AGE-BSA compared with the BSA control (n=4). **B**: Glycyrrhizin (100 μM), SP600125 (10 μM), and NAC (10 mM) decreased AGEs-induced upregulation of phospho-JNK2/3 (n=4). **C**: Treatment with GZ and NAC for 24 h showed insignificant changes relative to sterile PBS in cell viabilities of RGC-5 assayed with XTT (n=6). Abbreviations: **A**, AGE-BSA; **B**, BSA; G_50_ and G_100_, glycyrrhizin at 50 and 100 μM; SP, SP600125; N_5_ and N_10_, NAC at 5 and 10 mM.

HMGB1 and RAGE activate MAPK signaling pathways [26-28]. In the present study, we focused on activation of MAPKs by HMGB1 in RGC-5 cells incubated with AGE-BSA or BSA for 1.5 to 4.5 h. The results showed that only phosphorylated JNK2/3, but not phosphorylated p38, ERK, or JNK1, increased significantly at 3 h after treatment with AGE-BSA (Figure 5A). The timing of the JNK2/3 activation was consistent with the release of HMGB1 from AGE-BSA–treated RGC-5 cells in the supernatant. GZ (100 μM), which binds and inhibits the cytokine activities of HMGB1 [29,30], SP600125 (10 μM), and NAC (10 mM), successfully blocked the increase of phosphorylated JNK2/3 in RGC-5 cells treated with AGE-BSA for 3 h (Figure 5B). Treatment with GZ (100 μM), NAC (5 mM), and NAC (10 mM) for 24 h showed insignificant changes in RGC-5 cell viability on the XTT, which were 94.6%±5.4%, 97.9%±1.1%, and 98.8%±0.7% relative to control (sterile PBS; p=0.134, p=1.000, and p=1.000, respectively, Figure 5C).

### Advanced glycation end products induced vascular endothelial growth factor-A upregulation is high-mobility box 1 protein dependent and works through the c-Jun N-terminal kinase pathway

**Figure 6:**
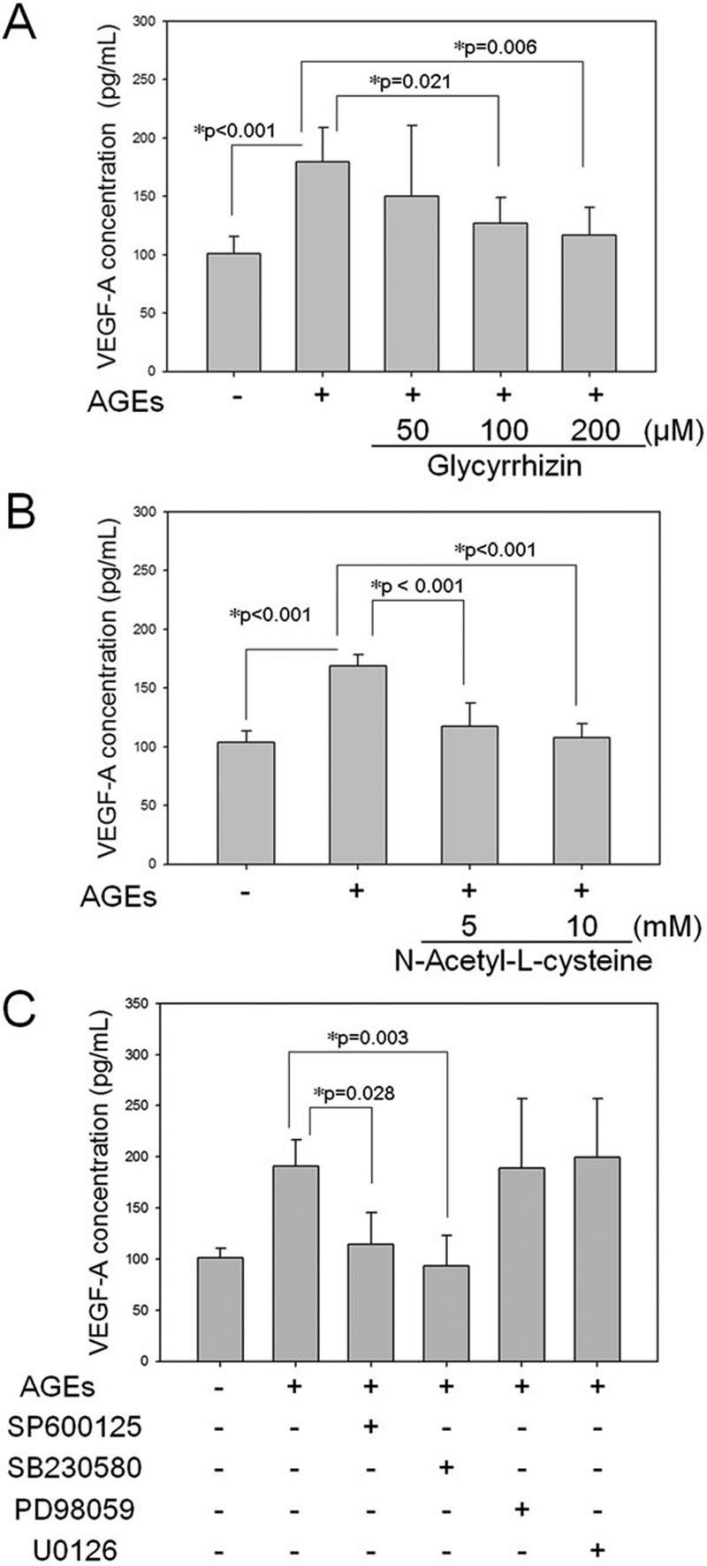
Advanced glycation end products–BSA (AGE-BSA) induced vascular endothelial growth factor (VEGF)-A expression is modulated by glycyrrhizin, antioxidant N-acetyl-L-cysteine (NAC), or specific c-Jun N-terminal kinase (JNK) inhibitor in retinal ganglion cell (RGC)-5 cells. Both glycyrrhizin (**A**) and NAC (**B**) significantly decreased the AGE-BSA-induced increase of VEGF-A concentration in the culture media of RGC-5 cells (n=8). (**C**) AGE-BSA-induced increase of VEGF-A concentration in the culture media of RGC-5 (n=8), was significantly decreased by SP600125 (a specific JNK inhibitor), but not SB230850 (5 μM; p38 inhibitor), as well as PD98059 and U0126 (10 μM each; extracellular-signal-regulated kinase [ERK] inhibitors).

We used the HMGB1 inhibitor GZ to block HMGB1 and found a dose-dependent decrease in VEGF-A concentration in the culture media at 24 h after the RGC-5 cells were treated with AGE-BSA. GZ doses of 50, 100, and 200 μM suppressed VEGF-A concentration elevation by 38.0%±77.6%, 67.2%±28.1%, and 79.8%±30.0%, respectively (p=0.831, p=0.021, and p=0.006, respectively, Figure 6A). However, NAC at doses of 5 and 10 mM also inhibited VEGF-A concentration elevation by 79.0%±18.6%, and 94.1%±11.2%, respectively (p<0.001 and p<0.001, respectively, Figure 6B). Furthermore, specific inhibitors of MAPK signaling pathways, namely, SP600125 (10 μM) for JNK, SB230580 (5 μM) for p38, PD98059 (10 μM), and U0126 (10 μM) for ERK, were studied. Pretreatment of RGC-5 cells with these inhibitors before AGE-BSA treatment resulted in a decrease in VEGF-A concentration in the supernatant by 15.8%±72.6%, 95.2%±26.9%, −0.2%±89.1%, and −9.8%±74.8%, respectively (p=1.000, p=0.015, p=1.000, and p=1.000, respectively). The results showed that only SP600125, the specific inhibitor of the JNK pathway, could inhibit the increase of VEGF-A at 24 h after incubation with AGE-BSA (Figure 6C).

## Discussion

Previous studies have confirmed the effects of AGEs on the pathogenesis of a variety of diseases, including retinal microangiopathy [31-35]. In this study, we confirmed a unique role played by HMGB1 in AGE-BSA-induced VEGF-A secretion from RGC-5 cells. Our results are consistent with recent studies that showed that HMGB1 provides a strong proangiogenic stimulus both in vitro and in vivo [12,13]. It has been demonstrated that

HMGB1 stimulates membrane ruffling and repair of a mechanically wounded endothelial cell monolayer, causes endothelial cell sprouting, and stimulates neovascularization of chicken embryo chorioallantoic membrane via RAGE [12]. The crucial role of HMGB1 has also been demonstrated in diabetic mice for ischemia-induced angiogenesis through a VEGF-dependent mechanism [13].

VEGF-A has been recognized as a critical mediator of diabetic retinopathy in experimental and clinical studies [34-36]. However, the modulation of VEGF-A in the retina of diabetic patients before the stage of proliferative retinopathy with neovascularization is not completely clear. We have demonstrated that HMGB1 might play a role in the upregulation of VEGF-A in retinal ganglion cells after exposure to AGEs. Binding of glycyrrhizin to HMGB1inhibits the phosphorylation and physiologic activities of HMGB1 in vitro [29]. Glycyrrhizin has also been reported to inhibit the chemoattractant and mitogenic activities of HMGB1, and only has a weak inhibitory effect on its intranuclear DNA-binding function [30]. In our study, blocking HMGB1 with glycyrrhizin successfully inhibits AGE-BSA-induced upregulation of VEGF-A.

Blocking HMGB1 suppressed the AGEs-induced upregulation of VEGF-A in our study. We deduce that the extracellular HMGB1 protein works as a cytokine or a cofactor that amplifies the effect of the AGE-RAGE axis, in an autocrine/paracrine manner, and mediates the secretion of survival factors including VEGF-A for counteracting the oxidative stress. Glycyrrhizin used in our study suppressed 38.0% to 79.8% of VEGF-A upregulation in response to AGEs, instead of totally suppressing it; therefore, a reduced level of VEGF-A upregulation and secretion from RGC-5 cells was induced by AGEs without augmentation of HMGB1. Another possible explanation for the effect of glycyrrhizin on suppression of VEGF-A upregulation is the other signaling pathway triggered by HMGB1 outside the AGE-RAGE axis. In our study, an increase in the TLR4 protein level was detected in RGC-5 cells treated by AGEs for 24 h. HMGB1 can form highly inflammatory complexes with single-stranded DNA, lipopolysaccharide, interleukin-1beta, and nucleosomes, which interact with TLR9, TLR4, interleukin 1 receptor, and TLR2 receptors, respectively [37]. HMGB1 isolated from cells cultured in the presence of IL-1β, IFN-γ, and TNF-α had enhanced proinflammatory activities through binding to mediators such as IL-1β [38]. One recent study showed that Toll-interleukin 1 receptor domain-containing adaptor protein and MyD88, which are known to be adaptor proteins for TLR2 and TLR4, bound to the phosphorylated RAGE after ligand binding and transduced a signal to downstream molecules [39]. TLR signaling pathways have been shown to mediate VEGF production following acute myocardial ischemia-reperfusion [40], and lipopolysaccharide-induced prostaglandin (PG)I2/prostacyclin receptor interaction in macrophage [41]. The role of TLRs in RGC-5 cells and VEGF-A upregulation is the direction of our future study.

HMGB1 is released from neural cells in response to stresses such as chemical ischemia, oxidative stress by hydrogen peroxide, and excitotoxicity by glutamate [42]. In this study, HMGB1 was detected in the cytosol of RGC-5 cells and was released into culture media within 3 h of treatment with AGE-BSA. The cytosolic HMGB1 in RGC-5 cells could be the result of translocation from nuclei. The immunofluorescence studies in our study showed a pattern of cytosolic translocation, which has been reported in murine macrophage-like RAW 264.7 cells treated with hydrogen peroxide [43]. The release of HMGB1 is an active process rather than a passive phenomenon from damaged nuclei or necrotic cells, as there are no significant changes in cell viability and cytotoxicity in RGC-5 cells treated with AGE-BSA. Active secretion of HMGB1 from the RGC-5 cell line has not been demonstrated before the present study. However, the insignificant changes in HMGB1 from nuclear fraction in our study suggest that secretion of newly synthesized HMGB1 protein by RGC-5 may also be possible, which needs to be clarified with further studies.

The elevation of levels of intracellular ROS following AGE-BSA treatment could be responsible for triggering active secretion of HMGB1 from RGC-5 cells. BSA is commonly used as a control for evaluating the effect of AGEs in experimental researches such as in vitro study for diabetic nephropathy with cultured mesangial cells [5], and proximal tubular cell injury via peroxisome proliferator-activated receptor-gamma activation [33]. However, the generation of ROS is a potential drawback of using BSA for in vitro studies. BSA in a concentration of 10 mg/ml induced endoplasmic reticulum stress, and stimulated the production of cellular ROS in renal proximal tubule cells [44]. Although the concentration of BSA used in our study is only 1/50 of that used for renal proximal tubule cells, an increase of ROS was still observed in the BSA-treated RGC-5 cells. In our study, the AGE-BSA-induced ROS levels were significantly higher than those of BSA alone at 3 h of treatment, indicating the effect of advanced glycation overrode BSA itself.

Various intracellular signaling pathways, including ERK1/2 and stress-activated protein kinase/JNK, have been shown to be activated downstream of HMGB1 in the study for scratch wound closure of HaCaT keratinocyte [27], and hippocampal dysfunction in single-Ig-interleukin-1 related receptor (SIGIRR)-deficient mice [28]. Interaction between AGEs and RAGE on the cell surface may also signal via p38 and other MAPKs. In this study, we found unique upregulation of phospho-JNK2/3, but not phospho-p38 or phospho-ERK1/2, at the time of HMGB1 secretion in RGC-5 cells treated with AGE-BSA. Blocking the JNK pathway with a specific inhibitor effectively reduces AGE-BSA-induced VEGF-A production by RGC-5 cells; this effect was not observed in blocking p38 or ERK1/2. These results suggest preferential involvement of the JNK2/3 pathway in AGEs-induced VEGF-A production; however, the molecular mechanisms underlying this phenomenon await clarification.

**Figure 7:**
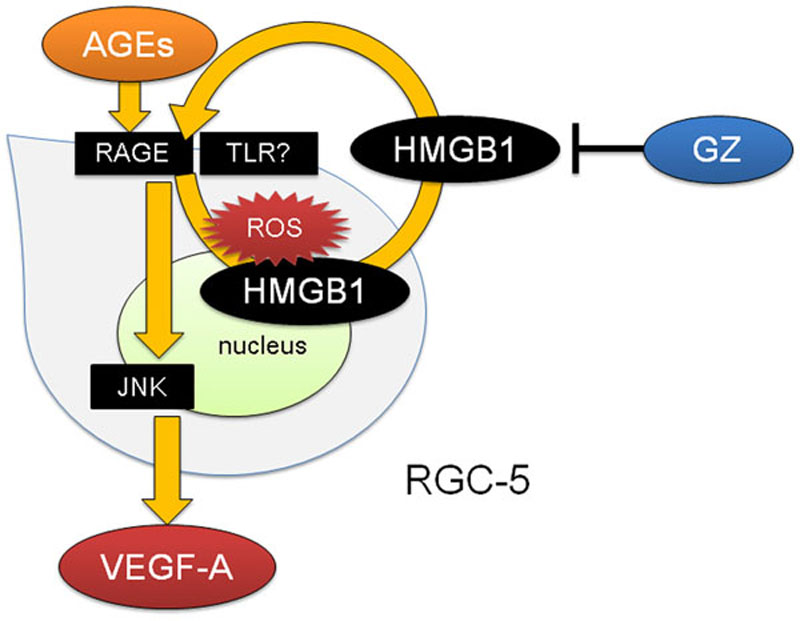
Hypothetic diagram of high-mobility group box protein 1 (HMGB1)-mediated upregulation of vascular endothelial growth factor (VEGF)-A in retinal ganglion cell (RGC)-5 cells in response to advanced glycation end products (AGEs) stimulation. The AGEs cause a rise in intracellular reactive oxidative species (ROS), which results in the release of HMGB1 into the extracellular space. Extracellular HMGB1 augments the signal via RAGE or TLR and mediates secretion of VEGF-A through the JNK signaling pathway, which was blocked by the HMGB1 inhibitor glycyrrhizin (GZ).

In conclusion, based on the results of this study, we propose that the HMGB1 protein plays a critical role in AGE-BSA-induced upregulation of VEGF-A in RGC-5 cells, which involves ROS production and the JNK signaling pathway (Figure 7). The findings from a single cell line study may not be sufficient to explain the pathophysiology of diabetic retinopathy involving the entire retina; however, we believe our results provide the framework for future studies investigating the role of HMGB1 in diabetic retinopathy.
